# Prevalence and determinants of Anemia among pregnant women in sub-Saharan Africa: a systematic review and Meta-analysis

**DOI:** 10.1186/s13690-021-00711-3

**Published:** 2021-12-03

**Authors:** Meseret Belete Fite, Nega Assefa, Bizatu Mengiste

**Affiliations:** 1 Department of Public health, Institute of Health Science, Wollega University, Nekemte, Ethiopia; 2 Department of Public health, School of public health, College of Health and Medical Sciences, Haramaya University, Harar, Ethiopia

**Keywords:** Prevalence of anemia, Intestinal parasite, iron and folic-acid supplementation, Systematic review, meta-analysis, Sub-Saharan Africa

## Abstract

**Background:**

Anemia is one of the world’s leading cause of disability and the most serious global public health issues. This systematic review and meta-analysis was carried out very prudently in order to give up the pooled prevalence and determinants of anemia in Sub-Saharan Africa.

**Methodology:**

To carry out this ephemeral systematic review and meta-analysis, a correlated literature review was done from various sources, PubMed Medline and Google Scholar Journals. Anemia related searching engine was used to make the study more evocative and intensive. We used modified Newcastle-Ottawa quality assessment scale for cross sectional studies to evaluate the quality of the study in relations of their inclusion. The Preferred Reporting Items for Systematic Reviews and Meta-Analyses (PRISMA) guideline was tracked to conduct this study. The pooled effect size was computed using the review manager and Compressive Meta-analysis software.

**Results:**

Twenty-fife studies, which encompassed 15,061 pregnant women, were chosen for the analysis. From those an overall prevalence of anemia in pregnancy in SSA was 35.6%. However, the result from meta-analysis showed that women who were infected with intestinal parasite were 3.59 times more likely to develop anemia compared to those who were not infected [OR:3.59, 95% CI (2.44,5.28)].The result showed that women who had no iron and folic-acid supplementation were 1.82 times more likely to develop anemia compared to those women who had iron and folic-acid supplementation {OR:1.82, 95% CI (1.22,2.70]. Women who had women were in third trimester pregnancy were 2.37 times more likely to develop anemia compared to those who were in first and second trimester [OR:2.37, 95% CI (1.78, 3.24)]. Women who had low dietary diversity score were 3.59 times more likely to develop anemia compared to those who had high dietary diversity score [OR: 3.59, 95% CI (2.44, 5.28].

**Conclusions:**

Our finding from this systematic review and meta-analysis displays the high case in prevalence of anemia among pregnant women in Sub-Saharan Africa. Predictors for this includes: intestinal parasite, iron and folic-acid supplementation, third trimester pregnancy and dietary diversified intake score were statistically correlated positively with anemia in pregnancy. These need cautious evaluation of impact of prevention effort for operational policy, programs and design nutrition intrusions for refining maternal food consumption during pregnancy. Also, dietary education intrusion requires to be prearranged to satisfy the desires of pregnant women. The finding of this work will be used as an evidences for policy makers of Africa; entirely for maternal and child health care. Lastly, we suggested further investigations to be carried out in the area of the study for more rigorous and comprehensive recommendations.

**Supplementary Information:**

The online version contains supplementary material available at 10.1186/s13690-021-00711-3.

## Introduction

In all over the world, anemia is one of the public health problems and continued as a universal top cause of frailty and the uppermost serious global health issues. This is because in a pregnancy, it is tremendously major both in industrialized and unindustrialized countries. Current suggestion from World Health Organization (WHO) document showed that, about 38% (32 million) of pregnant women are anemic in the word. Out of this, 46.3% (9.2 Million) of them are in Africa [1]. Nevertheless, the explanations of the rate frequently display inconsistency in the world from place to place [2].. For example, there is considerable variation in the rate of anemia during pregnancy within developed countries like United States in which the rate is 18%, in Australia 20%, in Singapore 67.8% and in China 70%; while the rates upsurge over trimesters [3–5]. However, the extent of the rate is becoming greater in developing countries; for instance, in Ethiopia 50.1%, in Sudan 53%, in Guinea 71% and in Pakistan 76.7%. These are the basic rationale problems associated to anemia, which is one of the fundamental concerns of public health issues in the world in over-all and in Africa in specifically [6–8].

Finding from a number of studies carried out on this present issue displayed that anemia in pregnancy has been related to adverse pregnancy outcome and fetal growth [9]. These includes premature birth, low birth weight, abortion, delay psychomotor improvement, impairment of cognitive recital and reduce totals on intelligence (IQ) test level of the newly born baby which has an effect on the later life of the children at all [10–16]. Furthermore, the impact of Iron Deficiency Anemia (IDA) in first stages of teenager and early youthful are not probably to be adapted by considerable iron administration [14]. This is because the iron dietary consumption upraises maternal mean hemoglobin concentration reads from 4.59 to 5.46 g/L. Therefore, excessive intake of dietary iron at first or successive trimester pregnancy is meaningfully associated with decrement of the threat of anemia. This results in decreases of adverse birth outcome, premature birth and LBW [6]*.* Equally, women in Sub-Saharan Africa (SSA) consume low dietary iron, Calcium and Folic-Acid having less than Recommended Dietary Allowances (DRA) requirements for a woman during pregnancy for the reason that they were economically not recognized [15, 16].

Various studies had examined multiple aspects upsetting anemia in pregnancy. The independent predictors which include maternal age, residence, literateness, antenatal care visit, inter-pregnancy interval, iron food consumption, dietary practice, micronutrient intake, dietary diversity, iron supplementation, parasite infection and gravidity were documented as factors associated with developing anemia in pregnancy [17–19]. The finding of the study suggested that women of third trimester pregnancy are more likely risky to develop anemia as compared to first and second trimester [20]. World Health Organization recommends day-to-day supplementation of 30–60 mg/d elemental iron (+ 400 μg) and folic acid to reduce the burden of anemia as a public health problem [1]. Finding of different studies also presented that compliance to Iron and Folic-Acid Supplementation (IFAS) in Sub-Saharan Africa countries has a better position to some degree. However the problem is silent leftovers at subnormal level in which compliance percentage arrays from 10.6% in Kenya to79% in Mozambique [21, 22].

A number of lately published studies on adherence with IFAS in pregnancy in Sub-Saharan African counties are recognized [23–47], but there is no systematic review and meta-analysis conducted on prevalence of anemia and its determinants in SSA. Furthermore, the present overall prevalence of anemia in pregnancy is not well-known in this setup empirically. Hence, in order to sum up studies carried out in different angles of SSA countries and give overall prevalence of anemia and its determinants; this systematic review and meta-analysis was done cautiously to alleviate the problem.

## Methods

To conduct this brief systematic review and meta-analysis, a related literature of articles from PubMed, Medline and Google Scholar journal data base were collected. To increase the probability of all-inclusiveness of the findings, uniterms and Boolean operators in English were used in searching strategies. Terms used for searching were: The search terms used were; “anemia OR anemia during pregnancy OR determinants of anemia” and name of African countries include: Kenya, Sudan, South Sudan, Ethiopia, Gabon, Nigeria, Ghana, Uganda, Benin, Somalia, Eretria, Malawi, Djibouti and so on. Finally, the results of this review were reported based on the Preferred Reporting Items for Systematic Review and Meta-Analysis statement (PRISMA) guideline.

### Selection of the studies

All articles related to prevalence and determinant of anemia were collected from various sources. Since the year of publication for each article were not restricted, all studies published up to February 25, 2020 were included for their eligibility in the review. Then, quantitative cross-sectional study design was used to make the study stronger and more meaningful. Nevertheless, studies published in qualitative methods were excluded due to the feature of the review and analysis selected to be used in this paper. Then to have a deep understanding of each article, all authors read the title and abstract part independently. To avoid biases, all eligible studies were screened and chosen after all individual’s full reading of the abstract section of each study. Then the disagreement of the work was managed to increase the reliability and validity of the review and analysis based on pre-set inclusion criteria. The reference lists of the studies screened for the systematic review and meta-analysis were surveyed to trace citations of references.

### Data extraction and quality assessment

All authors were participated in the data extraction. Data extraction template, which included author’s name, year of publication, study location, sample size, odds ratio, confidence intervals and *P*-value, was prepared before the extraction of data was carried out. After the extraction of the data by each individual independently, we made a cross checked and compared the results. All authors discussed and came to consensus on little partiality observed during the work. The Modified Newcastle-Ottawa quality assessment scale for cross-sectional studies was used to assess the quality of the studies in terms of its inclusion. The total score for the modified Newcastle–Ottawa scale for cross-sectional studies used was nine [9] stars as a maximum for the overall scale with the minimum of zero, and a study was considered to be a high quality if 7 was achieved from 9 and medium if 5 was achieved from 9 [48].

### Outcome interests

The primary outcome of this study was anemia in pregnancy period of the woman. Variables such as Age, Family size, income and parity were sought, but no sufficient information were obtained from published studies. Potential factors affecting the anemia includes: intestinal parasite, iron and folic-acid supplementation, third trimester pregnancy and Dietary Diversified Score (DDS). Thus, dietary diversity score was measured by collecting evidence on dietary consumption by means of 24-h dietary recall method. The score is classified as low (DDS < 3), medium (DDS = 4 or 5) and high (DDS > 5). Final clarification is the fourth visit for antenatal care which is defined as pregnant women who received antenatal care four or more times during the pregnancy period.

### Statistically analysis

The extracted data was copied to Microsoft excel to be exported to review manager version 5.3 and the compressive meta-analysis version 2 software for careful analysis. Accordingly, statistical description related to anemia and its determinants were performed. Sensitivity analysis recognized five articles that were considered outliers, distortion and have made over estimation in the pooled effect size measurement were removed and excluded from the final meta-analysis. When the required information was not presented in the articles, the authors of the studies were contacted with their email address. Risk of bias for each study was carefully assessed. Accordingly, the trim-and-fill test was used for crude association to identify the possible effect of absences of studies from meta-analysis of the overall pooled effect size. All selected articles for this study have reported the Odd rations and helped the bias come from the converting. Regarding of lessening the information bias, alternative encouraging way was the use of original investigations that gained information from reliable exposure and outcome measures, such as laboratory tests and clinical records, rather than self-reported information.

The existence of statistical heterogeneity and the publications bias were tested by funnel plot and empirically through Egger’s regression test. The degree of trustworthiness was contemplated. The heterogeneity of studies was computed using the I-squared statistic. In this process, 25% was signified as low, 50% moderate and 75% as high heterogeneity score. Subgroup analysis was executed by the study sub-region and study type (Community based and / or facility based). The effect of particular predictor’s variables which consist of: intestinal parasite, iron and folic-acid supplementation, third trimester pregnancy and dietary diversified Score and the result of the meta-analysis revealed forest pilot and Odd Ratio (OR) with 95% of CI.

## Result

### Studies selection

**Fig. 1:**
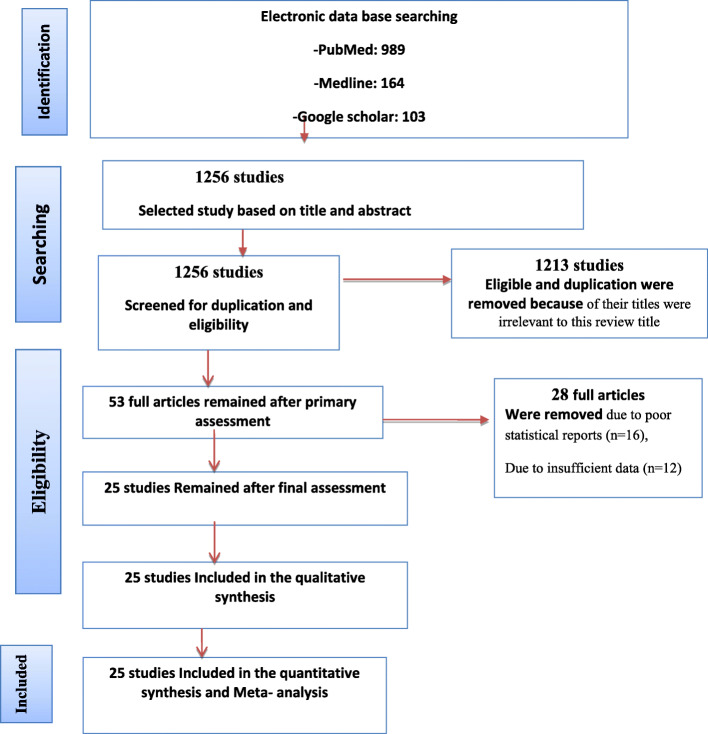
Flow diagram of the studies included in the Meta-analysis

Based on the objectives set for this work, we identified different studies related to the prevalence and determinants of anemia for the inclusion in meta-analysis before directly move to the other detail part of this paper. Accordingly, we found 1256 completed studies published on international journals. From these, 1313 of them were excluded for they were not satisfying the criterion of inclusion set in this study. However, 53 articles were chosen from those studies for their eligibility. Out of these 28 studies were rejected due to their poor statistical reports and defect of data observed in each of them. Finally, only 25 studies were added in this analysis for their neatness and clear justification. (Fig. 1).

### Characteristics of included studies

Twenty-three cross-sectional studies from different countries of Africa were included in the meta-analysi**s (23–47) (**Table 1**).** Out of those two of them (8%) were from Kenya, four (16%) from Ghana, two (8%) from Nigeria, two (8%) from Uganda, one (4%) from Benin, and fourteen (56%) from Ethiopia. Among those the highest sample size was observed in studies conducted in Benin [34] which was equal 3519 and the lowest was found in Kenya, 258 [47]. The mean age of the respondents was 27 years. Out of twenty-five studies incorporated in this review and analysis, though twenty-one studies **(24–31, 39, 41–47)** were conducted on facility based, four of them (**14, 23, 29, 31**) were done on community-based work**.**

**Table 1:** Characteristics of studies included in systematic review of prevalence compliance with IFAS among pregnant women in Sub-Saharan Africa

Author	Region	Study design	Study type	Sample Size	Prevalence in %
Addis Alene and A. Mohamed Dohe	Ethiopia	Community based	Community based	577	56.8
Adediran et al	Nigeria	Facility based	Facility based	170	46.6
Angesom Gebreweld and Aster	Ethiopia	Facility based	Facility based	228	11.6
Berhe et al	Ethiopia	Facility based	Facility based	304	7.9
Bolka and Gebremedhin	Ethiopia	Facility based	Facility based	349	31.5
Derso et al	Ethiopia	Facility based	Facility based	348	30.5
Freda Dzifa Intiful et al.,2016	Ghana	Facility based	Facility based	265	76
Getachew et al	Ethiopia	community based	community based	393	53.9
Grum et al	Ethiopia	Facility based	Facility based	634	16.88
Judith K. Anchang-Kimbi et al.,2016	Ghana	Facility based	Facility based	320	66.6
Judith Koryo Stephens et al	Ghana	Facility based	Facility based	316	41.5
Kefiyalew et al	Ethiopia	Facility based	Facility based	258	27.9
Lealem G. et al	Ethiopia	Facility based	Facility based	363	39.94
Lebso et al	Ethiopia	community based	community based	507	23.2
Mengist et al	Ethiopia			372	17.5
Nega et al.	Ethiopia	community based	community based	341	34.6
Niguse O et al	Ethiopia	Facility based	Facility based	374	36.6
Nonterah EA, Adomolga E, Yidana A et al	Ghana	Facility based	Facility based	506	42.7
O. T. Okube et al	Kenya	Facility based	Facility based	258	57
Obai et al	Uganda	Facility based	Facility based	743	22.1
Ononge et al	Uganda	Facility based	Facility based	2436	32.5
Ouédraogo et al	Benin	Facility based	Facility based	3519	68.2
Shitie et al	Ethiopia	Facility based	Facility based	284	2.8
Uneke C. J et al.	Nigeria	Facility based	Facility based	815	76.9
Wanjiru C.	Kenya	Facility based	Facility based	381	36.2

### Prevalence of Anemia

**Fig. 2:**
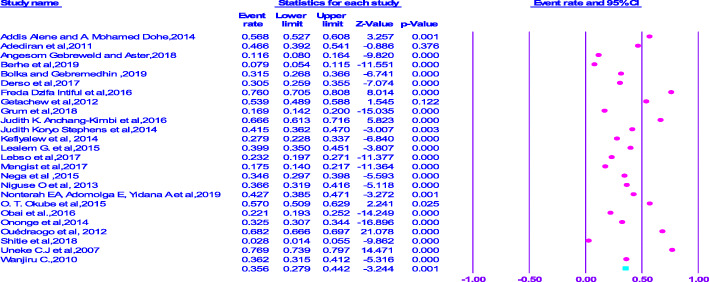
Forest plot displaying the pooled prevalence of anemia among pregnant women in Sub-Saharan Africa

From the analysis made, the lowest prevalence of anemia (7.9%) was observed in Ethiopia [25], but the highest compliance, which is equal to (76%) was observed in study conducted in Ghana [31]. On the other hand, the pooled prevalence of anemia amongst pregnant women in SSA was 35.6% (95% CI = 0.279–0.442) (Fig. 2).

### S**ubgroup analysis**

A subgroup analysis was done by classifying studies based on corresponding sub-regional location in Sub-Saharan Africa in order to compute and relate the prevalence of anemia focusing on athwart various participants’ characteristics. Based on this, the lowest prevalence of anemia in pregnancy was documented in Eastern Africa, (33.9%) (CI: 0.252, 0.438) and the highest prevalence of anemia was recognized in Western Sub-Saharan, (39.3(CI: 0.231, 0.582). However, a greater prevalence of compliance, which is equal to (41.4%%) was detected in studies conducted at facility level than community level (CI: 0.262, 0.584) (Table 2).

**Table 2:** Subgroup analysis of prevalence of aneemia among pregnant women in Sub-Saharan Africa

Subgroup	No. of included studies	Prevalence(95%CI)	Heterogeneity Statistics	Tau Squared	*P* value	I^2^	
By Sub- region							
Eastern Africa	17	33.9 (0.252,0.438)	1273.69	0757	< 0.000	98.85	
Western Africa	8	39.3 (0.231,0582)	877.78	0.856	< 0.000	98.35	
Overall							
By study type							
Facility based	21	41.4 (0.262,0.584)	2150.608	0.903	< 0.000	99.07	
Community based	4	34.5 (345,0.258)	97.35	0.389	< 0.000	96.91	

### Association of intestinal parasite with anemia

**Fig. 3:**
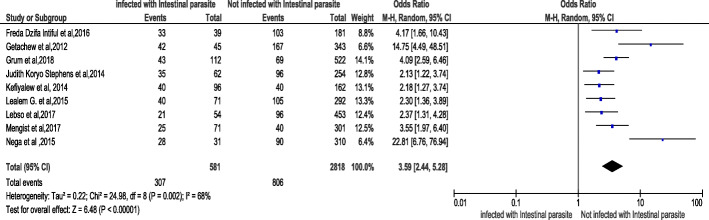
Forest plot displaying Association of intestinal parasite with anemia among pregnant women in Sub-Saharan Africa

Out of twenty-five chosen studies conducted on the area of the key concern and included in the meta-analysis, in nine of them (**31–39**), it was documented that infection of intestinal parasite was associated with anemia in pregnancy. Moreover, the result from meta-analysis also revealed that women who were infected with intestinal parasite were 3.59 times more likely to develop anemia compared to those who were not infected [OR:3.59, 95% CI (2.44,5.28)]. Thus, the heterogeneity test revealed I^2^ = 68% and the statistical evidence of this is *P* < 0.00001). From this we can understand that the random-effect analysis was the secondhand one. Thus, the Bag’s and Egger’s test for publication bias indicated that there is no statistical evidence of Publication bias. That is their *p*-values are equal to 0.117 and 0. 05 respectively (Fig. 3).

### Association of Iron Folate Supplementation with anemia

**Fig. 4:**
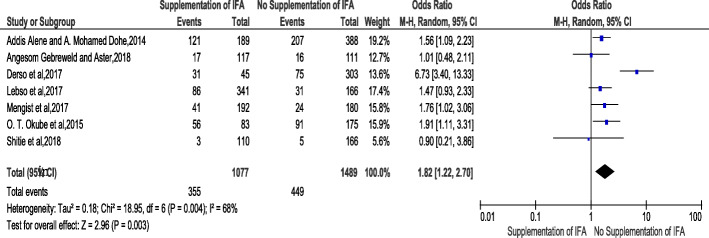
Forest plot displaying Association of Iron folate supplementation with anemia among pregnant women in Sub-Saharan Africa

The association of lack of iron folic-acid supplementation and risk of developing anemia during pregnancy was stated in seven chosen studies [23, 26, 29, 38, 39, 46, 47]. The result of meta-analysis from exhibited that women who had no iron folic-acid supplementation were 1.82 times more likely to develop anemia compared to those women who had iron folic-acid supplementation {OR:1.82, 95% CI (1.22,2.70] (Fig.4**)**. The heterogeneity test indicated (I2 = 68%) and statistical evidence of this heterogeneity was *P* < 0.003). Hence, the random-effect analysis was carried out. The Bag’s and Egger’s test for publication bias indicated that there is no statistical evidence of publication bias, which is equal to 0.76 and 0.85 (Supplementary 1).

### Association stages of pregnancy (trimester) with anemia

**Fig. 5:**
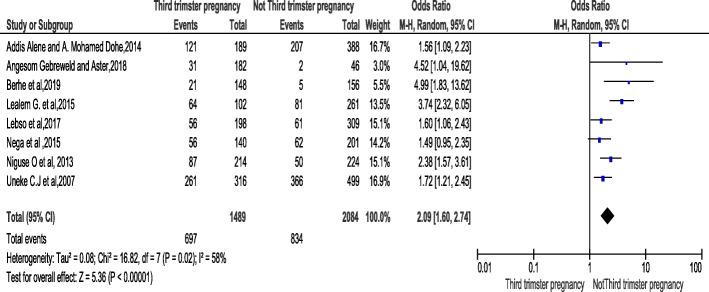
Forest plot displaying association of Pregnancy stage (trimester) with anemia among pregnant women in Sub-Saharan Africa

The important analysis was focus on the association of pregnancy stage and risk of developing anemia. This was stated in eight studies [23, 25, 26, 28, 37, 41, 42, 47]. The result of meta-analysis showed that women who were in third trimester pregnancy were 2.09 times more likely to develop anemia compared to those who were in first and second trimesters [OR:2.09, 95% CI (1.60, 2.74)}. The heterogeneity test indicated that (I^2^ is equal to 58% and the statistical evidence of this is *P* < 0.0001). Hence, the random-effect analysis was carried out. The Bag’s and Egger’s test for publication bias indicated that there is no statistical evidence of publication bias, which is equal to 0.17 and 0.12 respectively (Fig.5).

### Association of Dietary diversity score with anemia

**Fig. 6:**
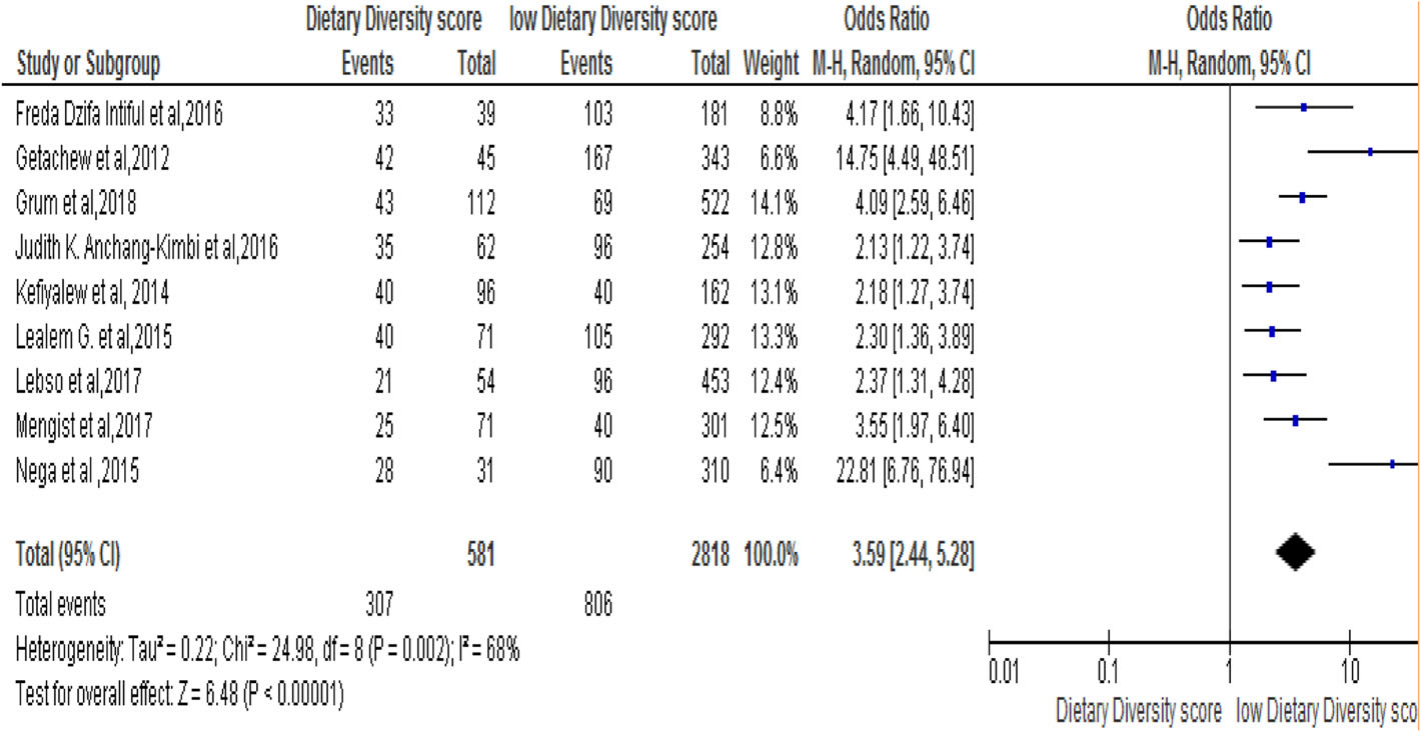
Forest plot displaying association of Dietary Diversity Score with anaemia among pregnant women in Sub-Saharan Africa

The association between Dietary diversity score and risk of developing noncompliance to IFAS during pregnancy was stated in eight studies [25, 28, 30, 31, 36, 37, 40, 43]. The result from meta-analysis from those revealed that women who had low Dietary Diversified intake Score (DDS) were 3.59 times more likely to develop anemia compared to those who had high DSS [OR: 3.59, 95% CI (2.44, 5.28]. The heterogeneity test indicated I^2^ = 68% and statistical evidence of this was < 0.00001). Therefore, the random-effect analysis was secondhand performed. The Bag’s and Egger’s test for publication bias indicated that there is no statistical evidence of publication bias, which is equal to 0.89 and 0.25 consecutively (Fig.6).

## Discussion

Anemia is one of the globally top causes of frailty, the highest universal problem and identified public health matters in Sub-Saharan Africa [1]. Evidences from the meta- analysis stated above suggested that almost 38% (32 million) women were victims of anemia in their course of pregnancy in the word.

The systematic review and meta-analysis presented in this paper showed the magnitude of anemia in sub-Saharan-Africa and its determinants. Accordingly, the key finding of analysis exhibited the anemia in pregnancy prevalence in Sub-Saharan Africa in which the pooled prevalence was equal to 35.6%; however, the heterogeneity test indicates its statistical evidence which was elucidated by difference in geographic location, for instance, Eastern Africa and western Africa types of study which was focused on community and facility-based type. In relation to this analysis, a study conducted in Iran stated that the prevalence of compliance with IFAS among pregnant women was 71.6% [47]. This systematic review and meta-analysis showed that; the magnitude of anemia among pregnant women in sub-Saharan-Africa and is determinants. Thus, the main finding of meta–analysis shown, anemia in pregnancy is rampant in Sub-Saharan Africa; in which the overall pooled prevalence of 36.6% was computed. Therefore, according WHO classification; anemia is moderate public health problem in Sub-Saharan Africa. Our finding differs substantially from studies conducted in developed countries; as the prevalence of anemia in Europe ranged 10–32% [17]. Compared to developed counties anemia is more prevalent in sub-Saharan Africa. This disagreement might be low socio-economic status of the region and poor dietary practice due to poor nutritional education intervention and irregular nutritional counselling in pregnancy.

It is evident that parasite infection reduces maternal hemoglobin level and contributes for higher incidence of anemia. The recent pooled meta-analysis showed that, women who were infected with intestinal parasite in their course of pregnancy were 3.59 times more likely risky to develop anemia compared to those with no history of parasite infections. Thus, this finding is comparably consistent with an investigation conducted in UK which documented that, parasite infection was significantly associated with anemia in pregnancy and one quarter of anemic mothers were infected with one or more intestinal parasite in their course of pregnancy [49]**.** Association of IFA supplementation was seen in this review. Women who were not supplemented with IFA in pregnancy were 1.82 times more likely risky to develop of anemia compared to their counterparts. An overflowing of studies had explored the effect of IFA supplementation on anemia. Iron use and high intake of iron was examined for the increased maternal haemoglobin and reduced risky of Iron deficiency at first and second trimester. Therefore, the finding of this study is comparably in line with different studies related to anemia which suggested that; women who are taking iron supplements have higher iron status and lower prevalence’s of anemia, which are dependent on the dose of iron and compliance [6, 17, 50, 51].

Anemia is a major cause of perinatal mortally and low birth in both developed and developing countries. Studies suggested that, 40% of all perinatal deaths are linked to anemia; whereas, worthy pregnancy outcomes occur 30–45% less often in anemic mothers, and their infants have less than one-half of normal Iron reserves [52]. Low birth weight contributes 60–80% of all neonatal deaths [53]. For every 10 mg increase in iron dose; birth weight increases by 15.1 g and risk of low birth weight decrease by 3% [6]. Therefore, supplementation of iron and folic acid is very important interventions for reduction of perianal mortality due to anemia. Our finding showed that, women of third trimester pregnancy were 2.37 times more likely risky to develop anemia compared to first and second trimester. In agreement to our finding, study conducted in china showed that the prevalence of anemia among pregnant women was higher during third **t**rimester (21.8%) than second trimester pregnancy (14.3%) [54, 55]. Nevertheless, a prospective pregnancy cohort study carried out in African-American confirmed that, pregnant women with depleted iron stores during the 2nd trimester were 12 times more likely to be classified with iron deficiency anemia compared to their 3rd trimester (56). Thus, this variation could be due to the parasite infection and inadequate dietary practice of pregnant women which is very common in a region. Delayed for intervention and IFAS might be another reason, since most of women in developing countries lately registered in anta natal care (almost second trimester).

The current review revealed that, women with low dietary diversified intake score were 3.58 times more likely risky to develop anemia during their pregnancy. Study investigated in Canada reported that, significant relationship was found between dietary diversity score and Haemoglobin level and iron intake among pregnant women and poor dietary habit and other lifestyle behaviour was positively associated with anemia in pregnant women [16].

However, the study lacks representativeness since there was no data found from some Sub-Saharan African counties. Also, there were no adequate studies incorporated in the analysis. Thus, this shortcoming could trouble the over-all prevalence of anemia among pregnant women in Sub-Saharan Africa.

### Strength and limitation

In this review, an extensive exploration method and more than one reviewer had taken part in all courses of review process. To do so, PRISMA guideline was carefully tracked throughout the review procedure. However, the analysis has its own defects because of a number of factors. Limitations include timing, duration and compliance of iron and folic acid supplementation which could have an effect on maternal anemia were not investigated in this study. Some Factors such as income, age and parity were not intensely examined. Other limitations of the current study are lacks of representativeness due to lack of similar studies from some of Sub-Saharan African counties and some studies have been omitted because of their poor statistical reports, their small sample size and their inadequate data. Researchers have contacted authors of the published studies but some of them did not make available some pertinent information to be considered for this review. Since we considered only the cross-sectional studies in the analysis, the outcome variable may possibly be affected by confounding variable and might affect the overall prevalence of anemia in pregnancy in Sub-Saharan Africa.

## Conclusion: implication for practice and future research

Our finding from this systematic review and meta-analysis displays the high case in prevalence of anemia among pregnant women in Sub-Saharan Africa. Predictors for this includes: intestinal parasite, iron and folic-acid supplementation, third trimester pregnancy and dietary diversity were statistically correlated positively with anemia in pregnancy. These need cautious evaluation of impact of prevention effort for operational policy, programs and design nutrition intrusions for refining maternal food consumption during pregnancy. Dietary education intrusion requires to be prearranged to satisfy the dietary desires of pregnant women.

Health care workers should have to offer dietary guidance to improve antenatal care service regularly. For instance, the recommendation from WHO reference presented that good supplementation needs making assurance that a pregnant woman is well prescribed for IFAS (lowest 90 tablets) and early registering her prior to 12 weeks of gestation period. The administration has to assign dietitian at all level of health system. Training should be programmed for health care workers who are at frontline antenatal care service at each level of health system in order to improve compliance of IFA supplementation at work place.

We hope that the finding of this work might be crucial as a bridging stone for policy makers of Africa; entirely for maternal and child health care. We suggested that further investigations to be carried out in the area of the study for more rigorous and comprehensive recommendations.

## Supplementary Information

**Additional file 1.** Supplementary file 1: Funnel plot displaying Publication Bias of Association of Iron folate supplementation with anemia among pregnant women in Sub-Saharan Africa. Description of figure: This figure presents, Bag’s and Egger’s test for publication bias showed no statistical evidence of publication bias.

## Data Availability

All data generated or analyzed during this study are included in this published article.

## Abbreviations

ANC: Antenatal care
WHO: World Health Organization
IFAS: Iron and Folic-Acid Supplementation
