# Supplementary material for: Prevalence and determinants of Anemia among pregnant women in sub-Saharan Africa: a systematic review and Meta-analysis
Source: Arch Public Health. 2021 Dec 3;79:219. doi: 10.1186/s13690-021-00711-3 (PMC8643002; doi:10.1186/s13690-021-00711-3)
Supplement: Supplementary file 1 — Additional file 1. Supplementary file 1: Funnel plot displaying Publication Bias of Association of Iron folate supplementation with anemia among pregnant women in Sub-Saharan Africa. Description of figure: This figure presents, Bag’s and Egger’s test for publication bias showed no statistical evidence of publication bias. [file 13690_2021_711_MOESM1_ESM.docx]

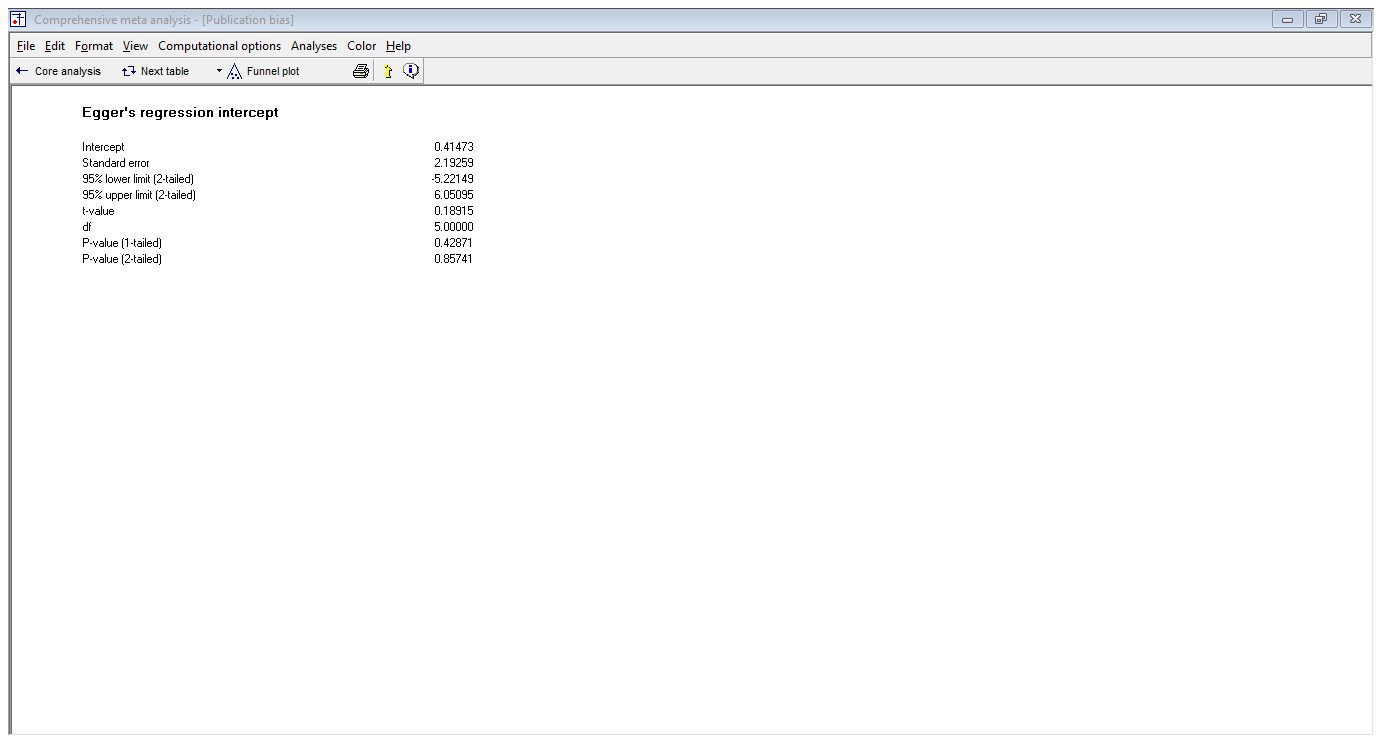

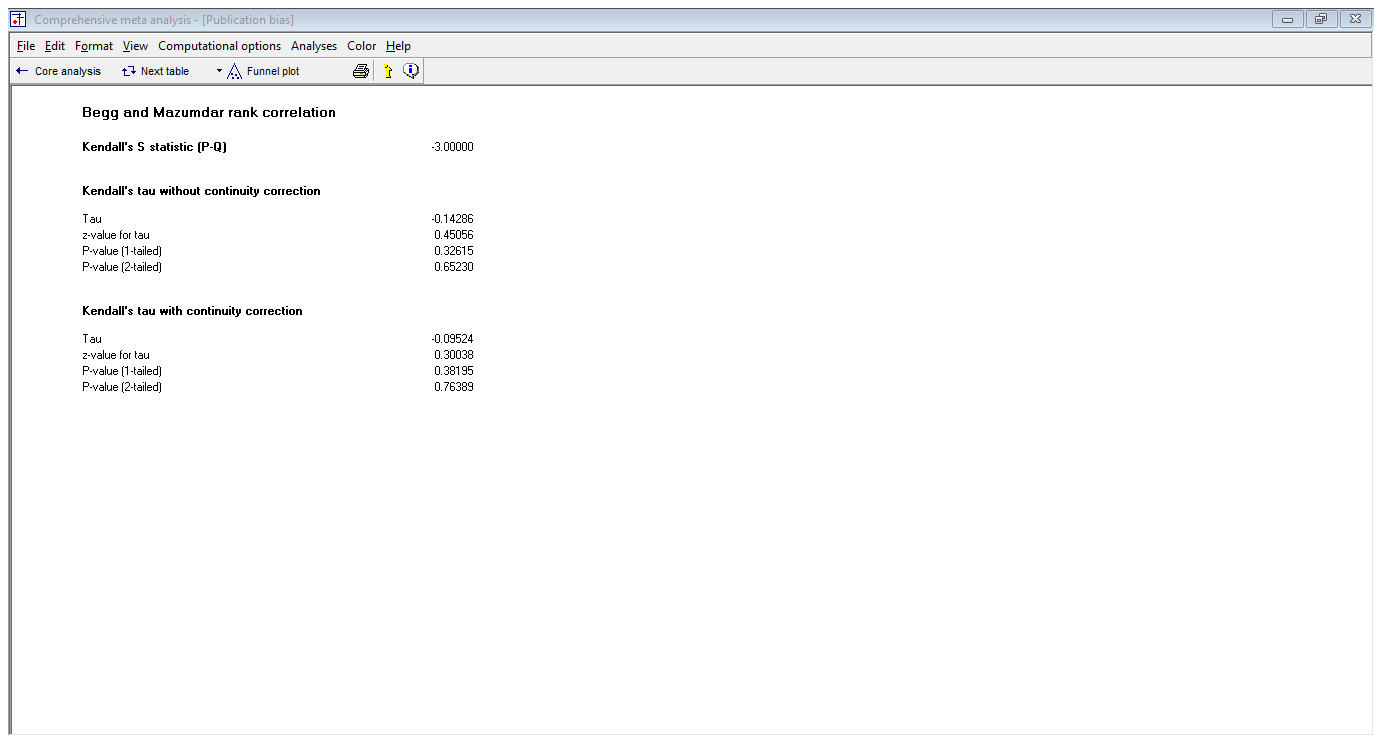


Supplementary file: 1: Funnel plot displaying publication bias of association of iron folate supplementation with anemia among pregnant women in Sub-Saharan Africa
